# Optimizing Antibiotic Prescribing for Acute Respiratory Tract Infection in German Primary Care: Study Protocol for Evaluation of the RESIST Program

**DOI:** 10.2196/18648

**Published:** 2020-09-30

**Authors:** Christin Löffler, Antje Krüger, Anne Daubmann, Julia Iwen, Marc Biedermann, Maike Schulz, Karl Wegscheider, Attila Altiner, Gregor Feldmeier, Anja Wollny

**Affiliations:** 1 Institute of General Practice Rostock University Medical Center Rostock Germany; 2 Institute of Medical Biometry and Epidemiology University Medical Center Hamburg- Eppendorf Hamburg Germany; 3 Association of Substitute Health Funds (Vdek) Berlin Germany; 4 National Association of Statutory Health Insurance Physicians Berlin Germany; 5 Central Research Institute of Ambulatory Health Care in Germany Berlin Germany

**Keywords:** antibacterial agents, respiratory tract infection, upper respiratory tract infection, lower respiratory tract infection, primary care, primary health care, physician-patient relation, shared decision making, antibiotic resistance

## Abstract

**Background:**

The emergence and increased spread of microbial resistance is a major challenge to all health care systems worldwide. In primary care, acute respiratory tract infection (ARTI) is the health condition most strongly related to antibiotic overuse.

**Objective:**

The RESIST program aims at optimizing antibiotic prescribing for ARTI in German primary care. By completing a problem-orientated online training course, physicians are motivated and empowered to utilize patient-centered doctor-patient communication strategies, including shared decision making, in the treatment of patients with ARTI.

**Methods:**

RESIST will be evaluated in the form of a nonrandomized controlled trial. Approximately 3000 physicians of 8 (out of 16) German federal states can participate in the program. Patient and physician data are retrieved from routine health care data. Physicians not participating in the program serve as controls, either among the 8 participating regional Associations of Statutory Health Insurance Physicians (control group 1) or among the remaining associations not participating in RESIST (control group 2). Antibiotic prescription rates before the intervention (T0: 2016, 1st and 2nd quarters of 2017) and after the intervention (T1: 3rd quarter of 2017 until 1st quarter of 2019) will be compared. The primary outcome measure is the overall antibiotic prescription rate for all patients insured with German statutory health insurance before and after provision of the online course. The secondary outcome is the antibiotic prescription rate for coded ARTI before and after the intervention.

**Results:**

RESIST is publicly funded by the Innovations funds of the Federal Joint Committee in Germany and was approved in December 2016. Recruitment of physicians is now completed, and a total of 2460 physicians participated in the intervention. Data analysis started in February 2020.

**Conclusions:**

With approximately 3000 physicians participating in the program, RESIST is among the largest real-world interventions aiming at reducing inadequate antibiotic prescribing for ARTI in primary care. Long-term follow up of up to 21 months will allow for investigating the sustainability of the intervention.

**Trial Registration:**

ISRCTN Registry ISRCTN13934505; http://www.isrctn.com/ISRCTN13934505

**International Registered Report Identifier (IRRID):**

RR1-10.2196/18648

## Introduction

### Background

Growing microbial resistance is a major challenge of all health care systems worldwide. The overuse and misuse of antibiotics in human and veterinary medicine accelerates this development [1,2]. In primary care, acute respiratory tract infection (ARTI) is the health condition most strongly related to antibiotic overuse [3,4]. This is a striking fact, as most cases of ARTI are caused by viruses and/or are self-limiting [5-7]. Considerable geographical variations in antibiotic prescribing for ARTI exist. In Europe, prescribing rates for ARTI range from below 40% in the Netherlands, Scandinavia, and Germany to up to 60% in the United Kingdom and Ireland, and reach up to almost 80% in southern Europe [8]. Reasons for inadequate prescribing include the physician’s overestimation of patient expectations toward antibiotic treatment as well as a deceptive “safety culture” in certain cases [9,10].

A considerable number of interventions to reduce inadequate prescribing for ARTI have been developed and evaluated. Overall, the main intervention strategies applied to date include laboratory and point of care testing (POCT), provider or patient education, computerized clinical decision support, prescribing feedback, delayed prescribing, and communication training. There is now sufficient evidence for the effectiveness of communication skills training and laboratory testing/POCT to reduce antibiotic prescribing for ARTI [11,12]. In addition, there is evidence that the effect of these interventions differs according to the level of antibiotic prescribing for ARTI in a given setting. High-prescribing countries benefit from laboratory testing/POCT, whereas medium- and low-prescribing countries show higher effects for communication training [12].

In parallel to the design and evaluation of interventional approaches, an increasing number of nations and supranational bodies have started initiating programs of antimicrobial stewardship. These usually include public information campaigns, education for health care providers, local surveillance of antibiotic resistance, and the provision of research grants to generate evidence necessary to improve antimicrobial stewardship [13]. Along with these initiatives, the German DART 2020 strategy was developed. Apart from strengthening a One Health approach to antimicrobial stewardship by addressing both human and veterinary medicine, supporting research is a major aim of the initiative. In this context, since 2016, the German *Innovationsfonds* granted several programs and trials addressing antimicrobial stewardship. The RESIST program is one of these programs. Within the program, approximately 3000 physicians of 8 (out of 16) German federal states can participate in online training aiming at patient-centered medical care for patients suffering from ARTI. Nationally and internationally, RESIST is one of the largest scientifically evaluated health intervention programs conducted to date. This protocol describes how the program is being evaluated.

### Objectives

RESIST aims at optimizing antibiotic prescribing for ARTI in German primary care. By completing a problem-orientated online training course, physicians are motivated and empowered to utilize patient-centered doctor-patient communication strategies, including shared decision making, in the treatment of patients with ARTI. Additionally, the courses provide current data on microbial resistance development and on rational antibiotic use. Moreover, physicians receive information materials for their patients as well as regional prescribing feedback. Based on this protocol, we are assessing whether the RESIST program reduces the number of antibiotics prescribed and, in the case of antibiotic use, impacts the choice of substance. The primary outcome measure is the antibiotic prescription rate for all patients insured with German statutory health insurance before and after provision of the online course. The secondary outcome is the antibiotic prescription rate for coded ARTI before and after the intervention. In addition, using individual qualitative interviews and focus group discussions, the perspective and perceptions of patients, physicians, and practice staff on the program will be evaluated.

## Methods

### Study Design

As RESIST is primarily a real-world project aiming to address as many participants as funding allows, it is evaluated in the form of a nonrandomized controlled trial. Patient and physician data are retrieved from routine health care data used by the Central Research Institute of Ambulatory Health Care in Germany (Zi). The Zi continuously analyzes prescription and diagnoses data on behalf of all German regional Associations of Statutory Health Insurance Physicians. The data analyzed include information on diagnosis as coded in practices and prescriptions as collected in pharmacies. Physicians not participating in the program serve as controls, either among the 8 statewide participating Associations of Statutory Health Insurance Physicians (control group 1) or among the federal states not eligible for participation in RESIST (control group 2). Antibiotic prescription rates before the intervention (T0: 2016, 1^st^ and 2^nd^ quarters of 2017) and after the intervention (T1: 3^rd^ quarter of 2017 until 1^st^ quarter of 2019) are compared.

### Recruitment

Recruitment of participating physicians is undertaken in 8 German federal states and regions (Bavaria, Baden-Wuerttemberg, Lower Saxony, North Rhine, Westphalia-Lippe, Brandenburg, Mecklenburg-Western Pomerania, and Saarland), assisted by the respective regional Association of Statutory Health Insurance Physicians. General practitioners, primary care pediatricians, and practice-based otorhinolaryngologists can enroll in the RESIST program on a voluntary “first-come, first-served” basis. The maximum number of participating physicians per region is related to the size of the regional association. In total, we aim to include up to 3000 physicians in the program.

### Participants

Physicians successfully completing the RESIST online training course are compared to nonparticipating physicians.

### Blinding

Participating physicians are not blinded as they enroll voluntarily in the RESIST program.

### Intervention

The intervention is based on an online training course consisting of three modules. Each module can be completed within approximately 1 hour. Module 1 focuses on patient-centered doctor-patient communication, and points to typical communicative situations when treating patients with ARTI. Within the module, physicians are encouraged to reflect upon these situations and to question perceived pressure to prescribe antibiotics. Modules 2 and 3 deal with rational antibiotic use in the case of upper and lower respiratory tract infections. After passing the Continued Medical Education–accredited course, physicians receive posters and brochures on rational antibiotic use to display them in their practices or to use them during consultations. Physicians successfully completing the course receive a one-time allowance. In addition, physicians receive further reimbursement for up to 20 patients with ARTI per quarter, which they document as treated according to the strategies adopted after participating in the online training. In addition, participating physicians receive a feedback report on aggregated data on regional antibiotic prescribing patterns. Figures are compared between intervention and control groups. See Multimedia Appendix 1 for more details on each component and module of the intervention [14].

### Control Group

Physicians not participating in the program serve as controls, either among the 8 participating regional Associations of Statutory Health Insurance Physicians (control group 1) or among the remaining associations not participating in RESIST (control group 2).

### Outcomes

The primary outcome measure is the overall antibiotic prescription rate of all patients insured with a German statutory health insurance before and after the provision of online courses. Secondary outcomes are (a) the antibiotic prescription rate for ARTI in patients aged ≥ 1 year before and after the intervention; (b) the antibiotic prescription rate based on different types of ARTI (eg, otitis media, bronchitis, pneumonia; see Table 1) before and after the intervention in patients aged ≥ 1 year; and (c) quality of antibiotic prescribing for ARTI measured by internationally accredited quality indicators of the European Surveillance of Antimicrobial Consumption Project (ESAC) [15], again before and after the intervention.

**Table 1:** International Classification of Diseases codes relevant for diagnosing acute respiratory tract infection.

Code	Diagnosis
J00	Acute nasopharyngitis (common cold)
J01	Acute sinusitis
J02	Acute pharyngitis
J03	Acute tonsillitis
J04	Acute laryngitis and tracheitis
J06	Acute upper respiratory infections of multiple and unspecified site
J09	Influenza due to identified zoonotic or pandemic influenza virus
J10	Influenza due to identified seasonal influenza virus
J11	Influenza, virus not identified
J12	Viral pneumonia, not elsewhere classified
J13	Pneumonia due to *Streptococcus pneumoniae*
J14	Pneumonia due to *Haemophilus influenzae*
J15	Bacterial pneumonia, not elsewhere classified
J16	Pneumonia due to other infectious organisms, not elsewhere classified
J17	Pneumonia in diseases classified elsewhere
J18	Pneumonia, organism unspecified
J20	Acute bronchitis
J21	Acute bronchiolitis
J22	Unspecified acute lower respiratory infection
J40	Bronchitis, not specified as acute or chronic
H65	Nonsuppurative otitis media
H66	Suppurative and unspecified otitis media

### Sample Size

With respect to the primary outcome, a reduction of the physicians’ overall antibiotic prescription rate by 1% is assumed for RESIST (from 33.8% in the control group 1 to 32.8% in the intervention group). Without consideration of cluster effects, 2 × 34,865 patients are needed to show this effect with a power of 80% and a type I error of 5% (two-sided). Assuming an intracluster correlation (ICC) of 8% [16] and an average cluster size of 160 patients per practice, a design effect of 13.72 is determined. Overall, the sample size increases to 2 × 478,400 patients (956,800 patients in total). Thus, 2 × 2990 practices (5980 in total) are needed for the evaluation.

For the secondary outcome, a reduction of 3% in the physicians’ antibiotic prescription rate for ARTI is expected (from 30.5% in the control group 1 to 27.5% in the intervention group). Assuming a power of 80% and a type I error of 5% (two-sided), 2 × 3590 patients are required. With an ICC of 8% [16] and an average cluster size of 127 patients (diagnosed with ARTI) per practice, the sample size increases to 2 × 39,751 patients (79,502 patients in total). Hence, 2 × 313 practices are needed (626 practices in total). The sample size necessary to investigate the effect of the intervention on the primary outcome will be adequate to investigate its effect on the secondary outcome as well.

### Data Collection, Completeness, and Quality

The trial is based on pseudonymized routine data provided by the Zi. The dataset is based on aggregated practice data. Information collected includes: age groups and sex of patients, ARTI diagnoses (grouped and individual diagnoses), antibiotic prescriptions, group affiliation of the physician (intervention vs control), region, level of urbanization, and type of medical insurance of the patient.

### Statistical Methods

Data analyses will be performed in accordance with the STROBE statement [17]. For descriptive statistics, we will report the primary and the secondary outcome for the whole sample and by treatment allocation. Sampling units are practices. This evaluation is designed to confirm the two-sided primary hypothesis that the physicians’ antibiotic prescription rate after T1, regardless of the diagnosis, is lower in the intervention group than in control group 1. For investigating the primary outcome, we will use a negative binomial regression with the absolute number of annual antibiotic prescriptions as the dependent variable, the number of patients as the offset variable, and the antibiotic prescription rate before the introduction of RESIST as a covariate. To avoid bias, we will include region, specialization of the physician, proportion of male patients, proportion of different age classes (≤7 years, 8 to 18 years, 19 to 65 years, >66 years), proportion of insurance status (member, pensioner, noncontributory dependents), and the interaction between the proportion of male patients and region as additional covariates. The comparison between the intervention group and control group 1 is considered in a confirmatory manner. The comparison between the intervention group and control group 2 is additionally examined as a sensitivity analysis. The model for the secondary outcomes will be formulated in the same manner. The results will be represented as incident rate ratios with their 95% CIs and *P* values. Statistical analyses will be carried out with SAS, version 9.4 or the newer version (SAS Institute, Cary, NC, USA).

With respect to quality indicators (according to ESAC), sampling units are practices. The analysis will be carried out in the same way as the analysis of the primary and secondary outcomes considering the requirements of each indicator.

### Process Evaluation

Qualitative interviews and focus group discussions will be used to investigate the perspective and perceptions of patients, physicians, and practice staff on the program. First and foremost, the qualitative evaluation focuses on factors fostering or hindering the implementation of the program. To this end, one to two focus group discussions per participating federal state or region will be conducted. Focus group discussions will include 6 to 8 physicians or practice staff members and are assumed to last about 60 to 90 minutes each.

To explore the patients’ perspectives, we will additionally conduct up to 50 narrative interviews by telephone. We will interview patients treated for ARTI by physicians participating in the RESIST program. We assume that phone calls will last about 10 to 30 minutes each.

Both focus group discussions and qualitative interviews will be recorded, transcribed verbatim, and analyzed based on content analysis [18].

To verify the findings of this qualitative process evaluation at large, a questionnaire for physicians and for patients suffering from ARTI will be developed. We aim to survey about 1000 individuals in each group. Physicians will be approached directly and will be rewarded monetarily for their support. Patient questionnaires will be distributed via participating physicians.

### Harms

The careful and sustainable prescription of antibiotics for ARTI is based on national and international clinical guidelines. Beyond that, the decision to prescribe antibiotics in the individual case remains the choice of the physician. Thus, harms are not expected.

### Study Registration

The program was retrospectively registered on December 6, 2018 at the ISRCTN Registry under reference ISRCTN13934505.

### Ethical Approval and Consent to Participate

The evaluation was approved by the ethics committee at Rostock University Medical Center in June 2017 (Reference: A 2017-0090). Participating physicians provide written informed consent. However, no informed consent is necessary from the patients, as pseudonymized and de facto anonymized routine data are used based on §80 SGB X.

### Availability of Data and Materials

The datasets generated and analyzed during the current study will be available upon request from the Zi (email: zi@zi.de) in the form of aggregated anonymized raw data collected from March 2020 to March 2025. Data will be shared upon request for the purpose of academic research and scientific analyses (such as meta-analysis).

## Results

RESIST is publicly funded by the Innovations funds of the Federal Joint Committee (G-BA) in Germany (funding code: 01NVF16005) and was approved in December 2016.

Recruitment of physicians is completed. A total of 2460 physicians participated in the intervention. Data analysis started in February 2020.

## Discussion

With up to 3000 physicians participating in the program, RESIST is among the largest real-world interventions aiming at reducing inadequate antibiotic prescribing for ARTI in primary care. Long-term follow up of up to 21 months will allow for investigating the sustainability of the intervention.

As RESIST utilizes an open-label nonrandomized evaluation concept, potential bias has to be carefully addressed. Bias may arise from the fact that physicians most interested in reducing inadequate antibiotic prescriptions are also likely to be those most motivated to take part in RESIST. To counteract this, we make use of an attractive incentivization and reimbursement scheme. In addition, the inclusion of comparable practices not participating in the program (both among participating regions and among those not participating in RESIST) allows for control of this potential source of bias.

We will also minimize bias due to changed coding. For example, a clinically identical patient treated with antibiotics might be coded with lower respiratory tract infection initially but with pneumonia after the intervention, leading to an overestimation of the interventional effect. By measuring the overall antibiotic prescribing rate of antibiotics without regard to any specific disease as the primary outcome measure, we can exclude bias due to coding phenomena.

Last but not least, the RESIST program joins the ranks of other current initiatives to reduce inadequate antibiotic prescribing. Thus, contamination effects between the projects are possible. However, by including a high number of physicians and practices among half of all German states, we believe to be able to address this potential source of bias adequately.

## Appendix

Multimedia Appendix 1 Components 1-5 of the intervention.

## Abbreviations

ARTI: acute respiratory tract infection
ESAC: European Surveillance of Antimicrobial Consumption Project
ICC: intracluster correlation
POCT: point of care testing
Zi: Central Research Institute of Ambulatory Health Care in German
