# Supplementary material for: Optimizing Antibiotic Prescribing for Acute Respiratory Tract Infection in German Primary Care: Study Protocol for Evaluation of the RESIST Program
Source: JMIR Res Protoc. 2020 Sep 30;9(9):e18648. doi: 10.2196/18648 (PMC7557437; doi:10.2196/18648)
Supplement: Multimedia Appendix 1 [file resprot_v9i9e18648_app1.docx]

Component 1 - Module 1 of the Online-Training-Course on Doctor-Patient-Communication.

| 1 Short Name | Online training course for physicians to promote patient-centered communication. |
| --- | --- |
| 2 Goal and rationale | Information on antibiotic prescribing patterns and antibiotic resistance in Germany and Europe is provided as an introduction. The main focus of the module is on exploring patient expectations and shared decision making with patients with acute respiratory tract infections. The educational objective for physicians is to carry out a convincing physician-patient communication. |
| 3 Materials | Video clips of re-enacted scenes in practices. Additional information as text/graphics. |
| 4 Procedures | Various situations, typical for general practitioners, pediatricians and ENT practices, are displayed. In addition, background information is provided. Physicians can access the online training via the vocational training platform of the Association of Statutory Health Insurance Physicians Safenet. In order to complete the course, physicians will have to pass an online test to receive a certificate. |
| 5 Providers of intervention | The eLearning is designed and implemented by an eLearning company possessing experience in the health care sector. It includes interactive and multimedia elements (video clips) which have been evaluated in former studies, now customized to the precise issues of the RESIST program. |
| 6 Mode of delivery | Single intervention. All physicians in the intervention group of RESIST can access the eLearning course. |
| 7 Location | The online training course is integrated in the vocational training platform of Association of Statutory Health Insurance Physicians Safenet. It is accessible via the physicians’ user-portal after a successful registration for the RESIST program. The previous registration ensures that only participating physicians of the intervention group can access the e-learning tool. |
| 8 Frequency | Module 1 of the online course lasts approximately 45 minutes. It can be accessed by physicians in the period from March 1, 2017 to December 31, 2017. The final exam has to be successfully completed before December 31, 2017 and the resulting certificate has to be submitted before January 1, 2018.  The online training course is accessible during the implementation period so that participating physicians have the possibility to refresh their knowledge. |
| 9 Planned tailoring | No |
| 10 Fidelity enhancement |  |

Component 2 - Module 2 and 3 of the Online-Training-Course on rational antibiotic prescribing in case of upper (2) and lower (3) acute respiratory tract infections.

| 1 Short Name | Online training course for physicians on rational antibiotic prescribing in case of upper and lower acute respiratory tract infections. |
| --- | --- |
| 2 Goal and rationale | The educational objective is to provide information on rational antibiotic therapy in case of upper and lower acute respiratory tract infections. |
| 3 Materials | Online training course (Interactive Slides). |
| 4 Procedures | Provision of information on rational antibiotic prescribing in case of upper and lower acute respiratory tract infections. Physicians can access the online training via the vocational training platform of the Association of Statutory Health Insurance Physicians Safenet. In order to complete the course, physicians will have to pass an online test to receive a certificate. |
| 5 Providers of intervention | Recommendations on antibiotic therapy in case of upper and lower acute respiratory tract infections based on “Wirkstoff Aktuell” of the “Association of Statutory Health Insurance Physicians” and the “Drug Commission of the German Medical Association (AKDÄ)”. |
| 6 Mode of delivery | Single intervention. All physicians in the intervention group of RESIST can access the eLearning course. |
| 7 Location | The online training course is integrated in the vocational training platform of Association of Statutory Health Insurance Physicians Safenet. It is accessible via the physicians’ user-portal after a successful registration for the RESIST program. The previous registration ensures that only participating physicians of the intervention group can access the e-learning tool. |
| 8 Frequency | Module 2 and 3 of the online course last approximately 45 minutes each. They can be accessed by physicians from 01.04.2017 to 31.12.2017. The final exam has to be successfully completed before 31.12.2017 and the resulting certificate has to be submitted before 01.01.2018.  The course is accessible during the implementation period. |
| 9 Planned tailoring | No |
| 10 Fidelity enhancement |  |

Component 3 - Decision Aid for physicians.

| 1 Short Name | Decision aid for physicians on rational antibiotic prescription in case of upper and lower acute respiratory tract infections. |
| --- | --- |
| 2 Goal and rationale | The brochure summarizes information on rational antibiotic therapy in case of upper and lower respiratory tract infections. |
| 3 Materials | Brochure |
| 4 Procedures |  |
| 5 Providers of intervention | Recommendations on antibiotic therapy in case of upper and lower acute respiratory tract infections are based on “Wirkstoff Aktuell” of the “NationalAssociation of Statutory Health Insurance Physicians” and the “Drug Commission of the German Medical Association (AKDÄ)”. |
| 6 Mode of delivery | Single intervention. All physicians in the intervention group of RESIST receive the brochure. |
| 7 Location | Provision of printed material for individual physicians by mail. |
| 8 Frequency | Provided at the beginning of the project and after the first half of the term. |
| 9 Planned tailoring | No |
| 10 Fidelity enhancement |  |

Component 4 - Information Material for Patients.

| 1 Short Name | Patient information material. |
| --- | --- |
| 2 Goal and rationale | The information material focuses on patients affected by respiratory tract infections and aims to raise awareness of antibiotics and resistance development. |
| 3 Materials | **Poster:** Illustrates health education messages for patients.  **Flyer:** Includes patient-friendly information on acute respiratory tract infections, antibiotics (ineffectiveness of antibiotics in case of viral respiratory tract infection, development of resistances) and recommendations to promote the healing process.  **Infozept** (two pages): The first page contains instructions/recommendations for patients in case of an acute respiratory tract infection (partly to be filled out by the physician). The second page contains instructions for the patient in case an antibiotic was prescribed (partly to be filled out by the physician). |
| 4 Procedures | The core messages are guideline-based and developed in agreement with the consortium partners. |
| 5 Providers of intervention | Physician, pharmacist, psychologist, journalist, advertising expert. |
| 6 Mode of delivery | Group intervention. |
| 7 Location | Provision of information material for the physicians by mail. The information material is meant to be displayed in the waiting room (e.g. flyer and poster) and/or discussed and handed out to the patient by the physician (flyer, Infozept). |
| 8 Frequency | Each physician receives the information material for the patients at the beginning of the project and again after the first half of the term. Practices can reorder material based upon their needs. The physician is meant to display the material in the waiting room and/or hand it out to the patients. |
| 9 Planned tailoring | No |
| 10 Fidelity enhancement |  |

Component 5 - Data-based feedback reports for physicians.

| 1 Short Name | Data-based feedback reports for physicians, reminder. |
| --- | --- |
| 2 Goal and rationale | Self-reflection by means of aggregated, data-based feedback reports for physicians.  The feedback report aims to recall the project and presents the improvements achieved so far. In addition, aspects with potential for improvement were identified in order to draw the physicians attention to these aspects. |
| 3 Materials | Feedback reports are based on the claims data (diagnosis and prescription data) of all participating physicians as well as those of the control. The reports are stratified by section and region of Association of Statutory Health Insurance Physicians. Individual reporting is not possible, as either practice, doctor and patient are pseudonymized. |
| 4 Procedures | Feedback-report is sent by mail and reaches the participating doctors at the beginning of the first quarter in 2019. The report should enable the reflection of one's own prescribing behaviour in comparison to one's own specialist group in the same region. |
| 5 Providers of intervention | Central Research Institute of Ambulatory Health Care in Germany (Zi). The Zi obtains and produces prescription data centrally for the associations of SHI physicians. The Zi has expertise in the consolidation and evaluation of prescription and diagnostic data for patients with statutory health insurance in Germany. |
| 6 Mode of delivery | Group intervention. |
| 7 Location | The feedback reports is sent to the physicians by mail. |
| 8 Frequency | One-time delivery (1st quarter of 2019). |
| 9 Planned tailoring | No |
| 10 Fidelity enhancement |  |
